# A Smartphone App for Supporting the Self-management of Daytime Urinary Incontinence in Adolescents: Development and Formative Evaluation Study of URApp

**DOI:** 10.2196/26212

**Published:** 2021-11-15

**Authors:** Katie Whale, Lucy Beasant, Anne J Wright, Lucy Yardley, Louise M Wallace, Louise Moody, Carol Joinson

**Affiliations:** 1 Centre for Academic Child Health Bristol Medical School University of Bristol Bristol United Kingdom; 2 Evelina London Children’s Hospital Guy’s and St Thomas’, NHS Foundation Trust London United Kingdom; 3 School of Psychological Sciences Faculty of Life Sciences University of Bristol Bristol United Kingdom; 4 School of Psychology University of Southampton Southampton United Kingdom; 5 Faculty of Wellbeing, Education, and Language Studies, The Open University Milton Keynes United Kingdom; 6 Centre for Arts, Memory, and Communities, Faculty of Arts and Humanities, Coventry University Coventry United Kingdom

**Keywords:** incontinence, urinary incontinence, digital intervention, child health, pediatric, pediatric incontinence, smartphone, intervention development, mobile phone

## Abstract

**Background:**

Daytime urinary incontinence (UI) is common in childhood and often persists into adolescence. UI in adolescence is associated with a range of adverse outcomes, including depressive symptoms, peer victimization, poor self-image, and problems with peer relationships. The first-line conservative treatment for UI is bladder training (standard urotherapy) that aims to establish a regular fluid intake and a timed schedule for toilet visits. The success of bladder training is strongly dependent on good concordance, which can be challenging for young people.

**Objective:**

This paper aims to describe the development of a smartphone app (URApp) that aims to improve concordance with bladder training in young people aged 11 to 19 years.

**Methods:**

URApp was designed by using participatory co-design methods and was guided by the person-based approach to intervention design. The core app functions were based on clinical guidance and included setting a daily drinking goal that records fluid intake and toilet visits, setting reminders to drink fluids and go to the toilet, and recording progress toward drinking goals. The development of URApp comprised the following four stages: a review of current smartphone apps for UI, participatory co-design workshops with young people with UI for gathering user requirements and developing wireframes, the development of a URApp prototype, and the user testing of the prototype through qualitative interviews with 23 young people with UI or urgency aged 10 to 19 years and 8 clinicians. The app functions and additional functionalities for supporting concordance and behavior change were iteratively optimized throughout the app development process.

**Results:**

Young people who tested URApp judged it to be a helpful way of supporting their concordance with a timed schedule for toilet visits and drinking. They reported high levels of acceptability and engagement. Preliminary findings indicated that some young people experienced improvements in their bladder symptoms, including a reduction in UI. Clinicians reported that URApp was clinically appropriate and aligned with the best practice guidelines for bladder training. URApp was deemed age appropriate, with all clinicians reporting that they would use it within their own clinics. Clinicians felt URApp would be of particular benefit to patients whose symptoms were not improving or those who were not engaging with their treatment plans.

**Conclusions:**

The next stage is to evaluate URApp in a range of settings, including pediatric continence clinics, primary care, and schools. This research is needed to test whether URApp is an effective (and cost-effective) solution for improving concordance with bladder training, reducing bladder symptoms, and improving the quality of life.

## Introduction

### Background

Daytime urinary incontinence (UI), which is the involuntary leakage of urine during the day, is common in childhood and is generally assumed to resolve with age. However, there is evidence from epidemiological studies that childhood UI often persists into adolescence [1-5]. For example, in a UK-based cohort study, 4.2% of females and 1.3% of males reported experiencing daytime UI at 14 years [6].

UI in adolescence is associated with a range of adverse outcomes, including depressive symptoms, peer victimization, poor self-image, and problems with peer relationships [7]. Key concerns of young people with continence problems include the perceived stigma of incontinence, fear of bullying and social isolation, adverse impacts on academic achievement, and difficulties in self-managing their continence problems at school (eg, restricted access to toilets during lessons) [8].

Most cases of UI in children and adolescents are functional (ie, with no underlying neurological, structural, or anatomical cause [9]), and the first-line conservative treatment is bladder training (standard urotherapy) [10,11]. Bladder training is a behavior modification intervention that aims to promote regular fluid intake throughout the day, establish a timed schedule for toilet visits (emptying the bladder every 2-3 hours), educate patients on how the bladder works and the causes of UI, and provide guidance on establishing optimal voiding behavior (eg, optimal toilet posture and relaxing the pelvic floor).

Bladder training can be an effective treatment for UI [12,13]; however, success is strongly dependent on good concordance with the timed schedule of toileting and drinking [14]. Concordance is challenging for many young people, strongly depending on their level of maturity, self-motivation, and ongoing support from clinicians [15]. Suboptimal clinical care experiences in young people with incontinence (eg, poor continuity in care, high rates of relapse, and treatment failure) diminish their belief in the success of treatments and add to their distress [15]. There is evidence that young people with UI have a strong desire to be involved in decisions about their treatment and to feel supported in self-managing their bladder symptoms [15]. Promoting the acceptance of chronic health conditions and the need for ongoing active management is linked to more positive coping strategies and greater treatment concordance [16-19]. There is some evidence that supplementing bladder training with a timer watch might aid concordance in children [11,20]; however, these watches can attract unwanted attention from peers. Our research with young people has highlighted the need to provide an age-appropriate self-management solution to help them manage their bladder symptoms [8,15]. This is further supported by the literature on self-management of other health conditions and the growing use of smartphone technology [21,22].

### Objectives

This paper describes the development of a smartphone app (URApp [23]) for young people, which aims to improve their concordance with bladder training. URApp was co-designed with young people and clinicians and incorporates theoretically underpinned behavior change techniques (BCTs) [24]. The development of URApp was informed by the Medical Research Council guidance for developing and evaluating digital interventions [25], which recommends the use of theory to inform intervention design and delivery [26,27]. There is evidence that embedding behavior change theory in health interventions increases their effectiveness, and interventions that incorporate more BCTs are more effective [28]. The development of URApp was also guided by the person-based approach (PBA) for developing behavioral health interventions [29]. The PBA involves in-depth qualitative research with the target user population at every stage of the development process to understand and accommodate their needs. The interventions are iteratively optimized to improve their acceptability and feasibility and make them engaging for users. The paper aims to provide an overview of the development of URApp, including its design, prototype development, and usability testing.

## Methods

### Overview

The development of URApp comprised 4 stages, as follows:

1. Review of current smartphone apps for UI
2. Participatory co-design workshops with young people with UI to gather user requirements for the app and to develop the wireframes
3. Development of the app prototype
4. User testing of the app prototype comprising in-depth qualitative research with young people and clinicians to explore their views of URApp

**Figure 1:**
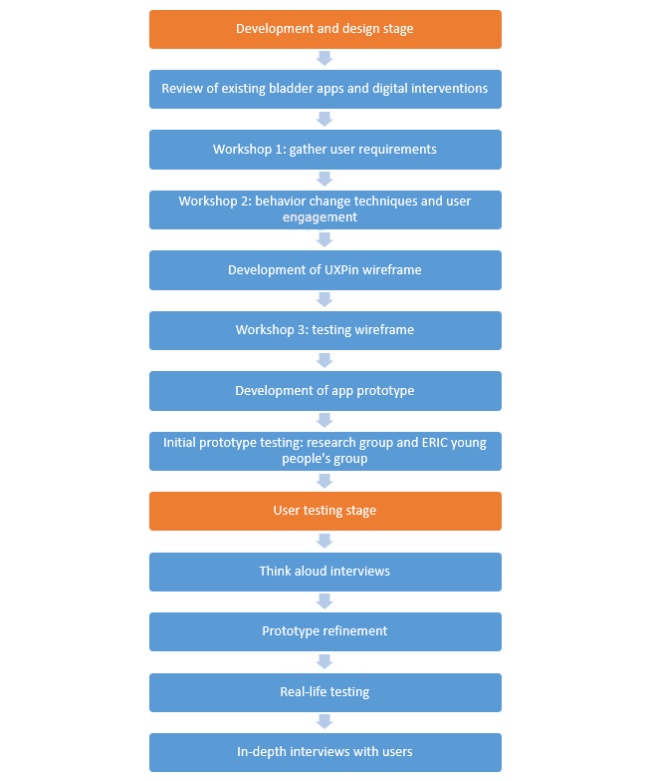
Flow diagram of the methods sequence.

The methods and results for each stage have been presented together to aid the understanding of the app development process. A flow diagram of the method sequence is shown in Figure 1.

### Ethical Approval

Ethical approval for all stages of the development process was granted by the University of Bristol research ethics committee.

### Clinical Input

Input from expert clinicians was obtained throughout the app development process to ensure that URApp was compatible with clinical advice given to young people receiving treatment for UI. Clinical input was obtained from (1) stakeholders in the study steering group, including a lead consultant pediatrician in charge of a specialist bladder clinic and a specialist bladder and bowel care nurse; (2) a clinical advisory group comprising 2 specialist nurses and a nephrologist; and (3) interviews with clinicians involved in continence care as part of the user testing stage.

### Inclusion Criteria

Participants were aged between 10 and 19 years, with current (or previous) experience of functional UI or urgency, able to provide informed consent (aged 16-19 years) or assent (aged 10-15 years), and able to speak and understand English. Young people taking part in user testing were also required to have an Android or iOS smartphone.

### Recruitment

Young people who took part in the participatory co-design workshops and user testing were recruited through advertisements on the website of ERIC, The Children’s Bowel and Bladder Charity [30], and the ERIC Facebook and Twitter pages. The advertisements provided an overview of the study and included links to allow potential participants (and their parents) to download the study information sheets.

Clinicians were recruited through an extensive network of clinical contacts established by ERIC and the Paediatric Continence Forum [31]. Purposive sampling was used to gain views of clinicians from a range of backgrounds, including continence nurses, pediatricians, urologists, and general practitioners.

### Consent and Assent

Written informed consent was obtained from all the participants. Parent consent and child assent were obtained from participants aged <16 years.

### Patient and Public Involvement and Advisory Groups

Patient and Public Involvement (PPI) in research is the research carried out *with* or *by* members of the public rather than *to*, *about*, or *for* them [32]. It can include patients, carers, and people who use health and social care services. A total of 2 PPI groups were formed to provide input for the running of the study and comprised 3 young people from the ERIC Young People’s Advisory Group and 3 clinicians (general practitioner, bowel and bladder nurse, and nephrologist). The clinician PPI group also provided feedback on the support pages in URApp to ensure that the information was consistent with clinical advice.

### Participants

The participants included 23 young people with current or previous UI or urgency. Table 1 provides a summary of participant characteristics and the phase of the development process in which they were involved. A total of 8 clinicians provided feedback about URApp in the qualitative interviews (Table 2).

**Table 1:** Demographic characteristics of the young people involved in the app development.

Participant ID	Age (years)	Gender	App development stage
W1	13	Female	Workshops 1, 2, and 3
W2	10	Female	Workshops 1, 2, and 3
W3	12	Male	Workshop 2
W4	10	Male	Workshop 3
W5	17	Male	Workshops 2 and 3
W6	12	Female	Workshop 1
W7	17	Female	Workshop 1
W8	15	Female	Workshops 2 and 3
W9	14	Male	Workshop 2
W10	12	Female	Workshops 1, 2, and 3
W11	11	Female	Workshop 3
P2	18	Female	TA^a^, RLT^b^, and IDI^c^
P5	11	Male	TA
P6	13	Male	TA, RLT, IDI
P8	19	Female	TA
P10	11	Male	TA, RLT, IDI
P11	18	Male	TA, RLT, IDI
P13	12	Female	TA, RLT
P14	14	Female	TA, RLT, IDI
P22	12	Female	TA, RLT
P23	11	Female	RLT, IDI
P26	12	Female	RLT, IDI
P27	11	Female	TA, RLT

^a^TA: think aloud.

^b^RLT: real-life testing.

^c^IDI: In-depth interview.

**Table 2:** Description of the professional background of the clinicians.

Participant	Role
Clinician 1	Clinical nurse specialist
Clinician 2	GP^a^
Clinician 3	Clinical nurse specialist
Clinician 4	School nurse
Clinician 5	Children’s specialist nurse
Clinician 6	Pediatric bowel and bladder care service clinical and professional lead
Clinician 7	Consultant urologist
Clinician 8	Clinical nurse specialist

^a^GP: general practitioner.

## Results

### Stage 1: Review of Current Digital Interventions to Support Young People With Daytime Wetting

#### Overview

In March 2017, we conducted a review of existing apps to ensure that none were specifically aimed at supporting self-management of bladder problems in young people. Existing apps were designed for young children and their parents to manage bedwetting, for pregnant or postpartum women (mainly for stress incontinence), and for older people with UI. Most apps provided only a bladder diary or reminders for pelvic floor exercises, and few were evidence-based or coproduced with stakeholders. This search was updated in April 2021, and no relevant apps were identified. A list of the reviewed apps is available on request.

#### Identifying the Core App Functions

We identified the core functions needed to support bladder training based on clinical guidance. Core app functions included setting a daily drinking goal, recording fluid intake and toilet visits, setting reminders to drink fluids and go to the toilet, and recording progress toward drinking goals. Clinicians advised that the app should also allow users to record stool frequency and consistency because of the comorbidity of constipation and UI [33]. A table outlining the key components of bladder training and target behavior change is included in Multimedia Appendix 1.

### Stage 2: Participatory Co-Design Workshops

#### Stage 2 Methods

Stage 2 focused on designing an app that supported the core features of bladder training. We invited young people to take part in 3 participatory co-design workshops at the University of Bristol to identify user requirements for the app (workshop 1), to explore which BCTs to use in the app to improve concordance (workshop 2), and to test an interactive wireframe created in UXPin (workshop 3) [34]. Wireframes provide a 2D blueprint of the app interface that allows testing of the app’s navigation and user journey and gain feedback on its content and layout.

The workshops were facilitated by researchers with expertise in health and developmental psychology, behavior change, qualitative research, participatory co-design, and user experience. All workshops had a lead facilitator and were guided by a detailed schedule of the content and structure for each activity. We used a range of tools to elicit the views of young people, for example, large (A3) phone templates for sketching ideas for app functions and screen layout and sticky notes with different colors and shapes to annotate the designs (see Figure 1 for example).

Before commencing the workshops, the research team had an initial meeting with the app development team (Natural Apptitude [35]) to discuss the purpose of the app and its core functions. The findings from each workshop were discussed with the app developers to ensure that the user requirements were feasible in terms of time and cost.

#### Stage 2 Results

##### Workshop 1: Identification of User Requirements and Implementation of Core Functions

**Figure 2:**
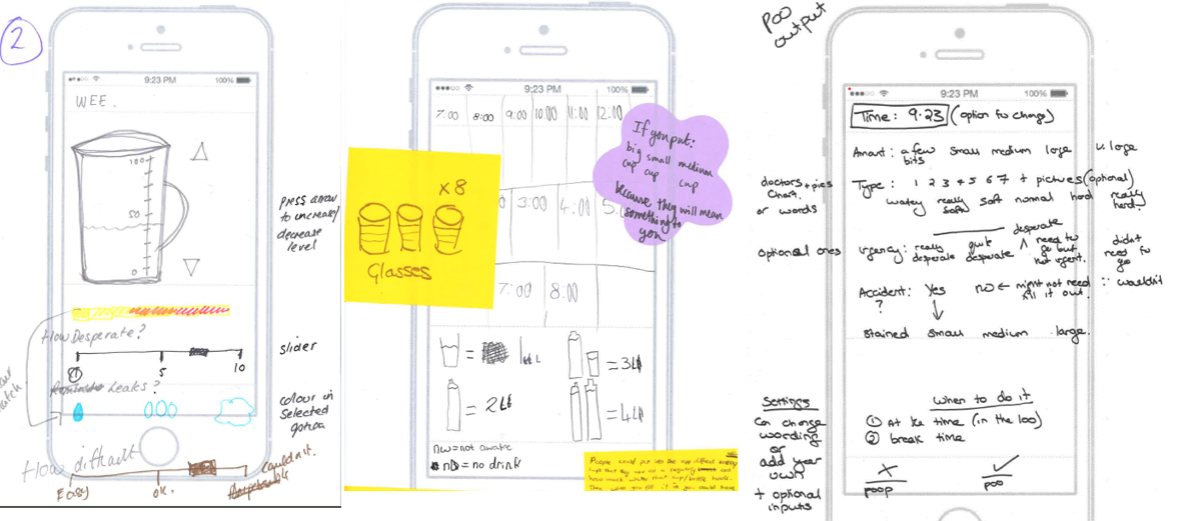
Workshop 1 example.

This workshop was led by a participatory co-design expert (LM) and a health psychologist (KW). The plan for the session was presented to the participants, and they were given a brief explanation of the key elements of bladder training. We asked the participants about their mobile phone use at home and at school or college, their preferred methods of recording toilet visits (wees and poos were their preferred terms) and drinking in the app, the potential ways to receive reminders for drinking and toilet visits, and information that would be useful to record in a daily diary (eg, mood, medications, and life events). Young people were also asked to provide feedback on the existing apps we had reviewed, and they reported that those apps did not meet their user requirements and were not age appropriate. Example results from workshop 1 are shown in Figure 2.

##### Workshop 2: User Engagement and Behavior Change

This workshop was led by a behavior change expert (LW). The first aim of this workshop was to identify how to maximize user engagement with the app. Activities included identifying apps that were popular among young people, discussing why they liked these apps, and examining the app functions that motivated their continued use.

The second aim was to obtain views on BCTs that could be implemented in the app to support self-management and improve concordance with bladder training. It was established that the app should provide rewards for changes in behavior within the young person’s control, that is, for recording their drinks and toilet visits and achieving daily drinking goals (recommended daily amount is 6-8 glasses of water or dilute squash regularly spaced throughout the day). The app would not provide rewards for fewer *wetting accidents* and *leaks,* as this could undermine motivation because of a perceived lack of personal control and competence [36].

**Figure 3:**
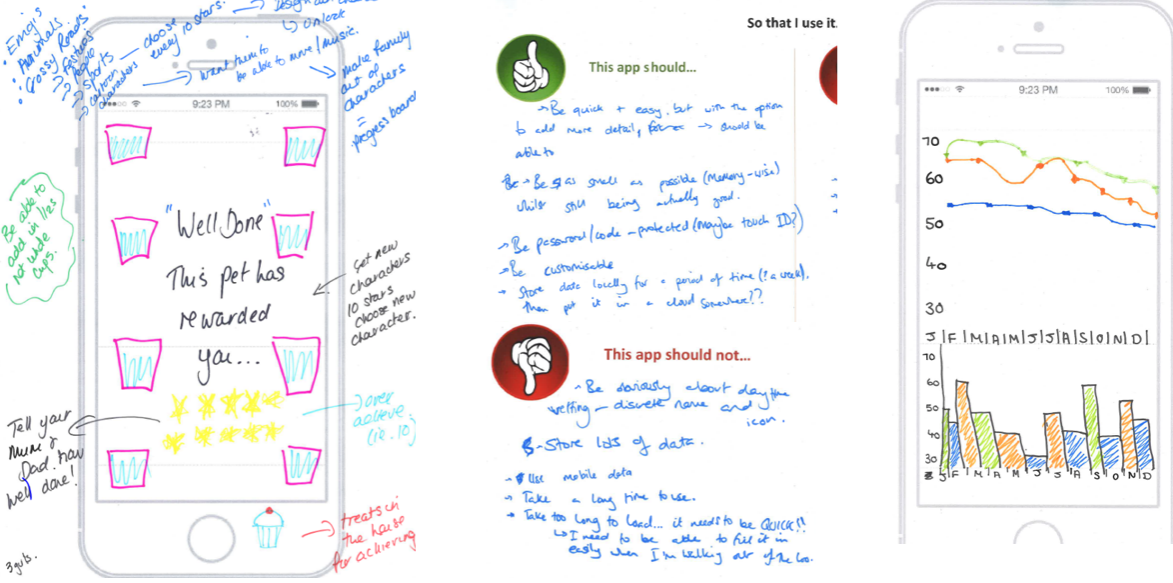
Workshop 2 example.

Participants provided ideas for daily rewards (eg, collecting stars and trophies) and *streak rewards* (for continuous daily use of the app, eg, used in Snapchat) that could help motivate them to keep using the app. They sketched ideas for recording progress toward daily drinking goals in the app (eg, progress bars and charts) and other data they wished to record (eg, number and type of daily toilet visits and number of wetting accidents). Figure 3 shows examples of workshop 2's outputs.

The workshop findings relating to BCTs were reviewed against the Behavior Change Taxonomy [24]. Optimal BCTs were identified as *graded goal setting and reviewing* (modifying goals to make them more achievable), *action planning* (ie, setting goals for daily drinking and incrementally increasing fluid intake), *prompts and cues* (ie, reminders to drink fluids and use the toilet), *rewards* (ie, for achieving drinking goals), and *self-monitoring* (ie, charts and a daily diary to review progress). The BCTs that were chosen in the app align with the self-determination theory [36] and are aimed at supporting users’ feelings of autonomy, competence, and relatedness, all of which have been shown to promote intrinsic motivation [37]. The *rewards* BCT further aligns with the theories of gamification [38,39]. Further input on the BCTs was gained from a digital intervention and behavior change expert (LY). The PBA to intervention design highlights the importance of responding to the user engaging with the app by providing personalized feedback [29]; therefore, this BCT was added to the URApp design. A full breakdown of the BCTs in URApp and how they are implemented is included in Multimedia Appendix 2. Following the first 2 workshops, an interactive wireframe was designed using UXPin. This is an essential stage in the design process and provides a visual guide for app layout, navigation between screens, and basic functionality.

##### Workshop 3: Feedback on the Wireframe

**Figure 4:**
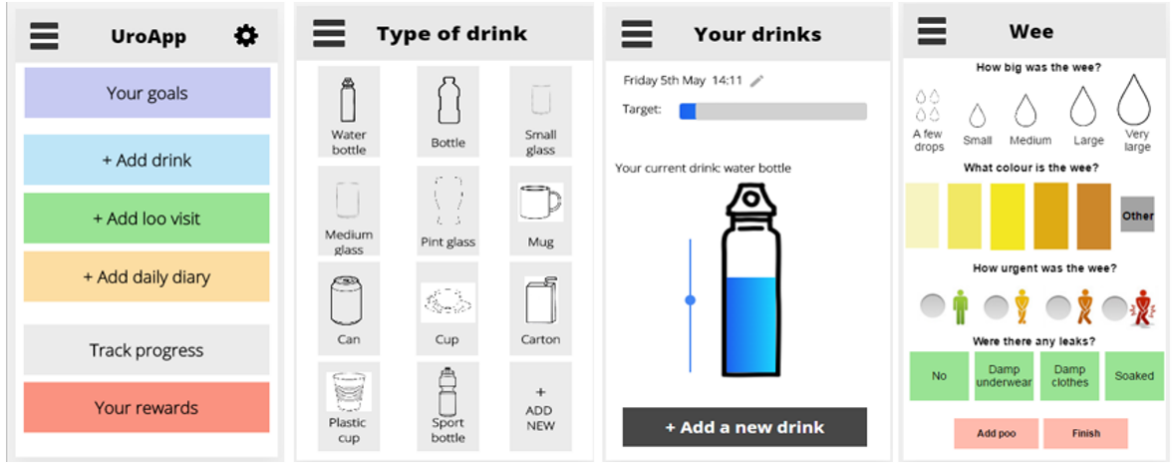
Wireframe example.

This workshop was led by an expert in user experience and prototyping (SC). The aim of workshop 3 was to review the interactive wireframe, design the app setup instructions and the drinks and reminders functions, and identify what information resources to include in the app. The wireframe was demonstrated (Figure 4) on a large screen. Participants were provided with smartphones and tablets to test the wireframe using a set of tasks aimed at navigating through the app screens and testing specific app functions (eg, adding a new drink). After completion of the workshop, adjustments were made to the wireframe based on user feedback (eg, changing *loo visits* to *visits* for more privacy, storing and reopening unfinished drinks, and a library of recent drink containers).

### Stage 3: Development of the App Prototype

#### Stage 3 Methods

Following completion of the workshops and adjustments to the wireframe, the research team collaborated with the app developers to produce the app prototype. A matrix was produced containing all the app functions, the purpose of each function, and how they should work. The priority of each function was defined using the MSCW (must have, should have, could have, would have) framework. Decisions were based on whether the function was deemed to be core or additional; participants’ and clinicians’ rating of importance; and the feasibility, technical difficulty, and cost of implementing each function in the app.

The developers shared regular videos to demonstrate their progress with the app build and designs for each screen and had regular meetings with the research team to review each stage of the build.

The initial app prototype was tested at a workshop attended by the ERIC Young People’s Advisory Group. They were asked to provide feedback on the app’s design, navigation, and functionality. This feedback was discussed within the research team, and the proposed changes were sent to the developers. Updated versions of the app were then made available to the research team for review through a closed (beta) testing group, and this iterative process continued throughout the app development process.

#### Stage 3 Results: Design and Content of Prototype

A prototype of the app was produced with the following functions:

- Passcode for security
- Daily drinking goal set by the user and based on clinical guidance
- Customizable reminders for drinking and toilet visits
- An interactive homepage to record drinks and view daily drinking progress
- A range of standard drink containers
- Option to add a customized container and use a picture of the user’s own container
- Toilet visit recording
- Progress charts, daily diary, and summary
- Rewards for reaching drinking goals
- Personalized feedback linked to the support and advice pages
- Task center for viewing notifications and completing tasks (eg, viewing daily feedback and completing weekly evaluations)

Example screenshots of the drink recording page, range of drinking containers, and toilet visit recording page are shown in Multimedia Appendix 3.

### Stage 4: User Testing

#### Stage 4 Methods

##### Think Aloud Interviews

Young people participated in *think aloud* interviews to provide their immediate reactions to every element of the app. This phase was key to optimizing the design and function of the app before real-life testing. Feedback from young people was logged in a *table of changes*, and the coding framework described in Table 3 was applied [29]. The table included positive and negative feedback on all functions of the URApp, suggested changes, reason for changes, and MSCW priority. Potential changes were discussed with stakeholders, and the decisions were communicated to the developers who implemented them before the real-life user testing.

**Table 3:** Table of changes for the coding framework.

Code	Full form	Meaning
IMP	Important for behavior change	This is an important change that is likely to affect behavior change or a precursor to behavior change (eg, acceptability, feasibility, persuasiveness, motivation, and engagement) or is in line with the logic model or with the guiding principles. For example, participants appear unconvinced by an aspect of the intervention, so you decide to add motivational examples.
EAS	Easy and uncontroversial	An easy and feasible change that does not involve any major design changes; for example, a participant was unsure of a technical term, so you add a definition.
REP	Repeatedly	This was said repeatedly by >1 participant.
EXP	Experience	This is supported by experience. Please specify what kind of experience; for example, patient and public involvement members agree this would be an appropriate change, and experts (eg, clinicians on your development team) agree that this would be an appropriate change. This is supported by evidence in the literature.
NCON	Does not contradict	This does not contradict experience (eg, evidence) or the logic model or the guiding principles.
NC	Not changed	It was decided not to make this change. Please explain why (eg, it would not be feasible or only one person said this).

##### Real-life User Testing

In the real-life testing phase, young people were provided with the updated app prototype to use for a period of 4 to 6 weeks. This is the minimum time needed to see improvements in bladder symptoms as a result of bladder training. At the end of the testing period, participants were invited to take part in an in-depth interview (on Skype) about their experiences of using URApp. The in-depth interviews were guided by a semistructured topic guide, which included sections on using URApp (general usability and function), BCTs included in the app, barriers to app use, health beliefs (understanding of bladder training and views on whether changes in drinking and toileting have affected symptoms), and views on using the app in consultations with clinicians (eg, specialist nurses and urologists). A deductive framework approach was used to analyze the interviews [40].

##### Interviews With Clinicians

We conducted semistructured interviews with clinicians involved in continence care to gain their feedback on the app design and function, clinical use and appropriateness, and potential implementation within clinical care. Clinicians were guided through the initial app setup and functionality of the app. Specific feedback was sought on whether the app functions aligned with the best practices in bladder training guidelines. Interviews were conducted by phone because of clinician location and availability.

#### Stage 4 Results

##### User Feedback

Of the 23 young people, 10 (43%) took part in the think aloud interviews, and 10 (43%) took part in the real-life testing (see Table 1 for full details). Young people reported that they liked URApp’s appearance and functions and thought that it could be helpful for self-managing their bladder symptoms:

It’s easy to use, it’s not confusing, the setup, the layout...I think I would use it. [P22]Much better than paper diaries...I never got around to filling them out, whereas this would be on my phone, which I would have on me, so I think that’s a lot handier.

P8

##### App Design

Participants found the design of the app pleasing and suitable for a wide range of ages. Younger participants said that they would like to have the option to customize the app and make it more personalized to them:

It looks good, they’re [the design graphics] simple, you don’t want them too flashy because it might take away from the actual purpose. [P2]I think it needs more personalisation, because it’s something you’re going to go on quite a lot so you want it to be personal...so you could change the colours, or add photos, pictures.

P15

##### Core Functions

The core function of the app, recording drinks and toilet visits, worked well. Users liked how quick it was to record toilet visits and the interactive nature of recording drinks by pulling down the fluid level with their fingers. However, some participants felt that this function needed to be made clearer:

You can drag by moving the water...Maybe make it a bit more obvious, I only knew because I accidentally touched it and it moved...maybe when you first download the App have a walk through. [P2]Oh yeah you just drag it...Oh that’s cool! You drink it yourself! You do a virtual drinking, that’s so cool! And you get stars! This is a brilliant app!

P10

Feedback highlighted that the ability to add drinks retrospectively should be made clearer:

It wasn’t obvious how to change the time of the drink...maybe you could make it bigger?

P14

Young people liked the toilet visit options and found the choices clear and with nice graphics. Two areas for improvement were highlighted: first, including *wee leak* options for incontinence pad wearers (ie, wet pad), and second, more information on how to judge stool consistency.

##### Data Display

Data collected in URApp are displayed in 3 ways—a progress chart (line and bar graph), a daily diary, and a summary providing an overview for the chosen period (eg, wees have mostly been large). Feedback on how the data were displayed varied between participants; for example, older participants tended to like the progress charts and found them helpful in identifying patterns and tracking changes over time:

It’s easier to spot patterns, you don’t have to work it out for yourself. [P8]You can change what data is shown, it’s another nice way of being able to see. [P2]I think it’s a good idea, you can see how much you’ve improved, if the app is helping you.

P22

Other participants preferred the summary and daily diary, as they provided a simpler overview of the data. The key feedback was that users wanted a space to record events, their mood, and any factors that might affect their symptoms, such as stressful events, changes in medication, or certain drinks:

Being able to track things using your own words as well and maybe being able to track your mood, because it’s something that’s closely linked to having a bladder or bowel condition.

P2

##### Rewards and Feedback

Feedback on the rewards was very positive, with young people reporting that they liked all the streaks, stars, and trophies. Younger participants particularly liked the star and trophy rewards and thought this would encourage them to use the app and keep them motivated:

It makes you feel more committed to achieving your goals...You achieve stuff...It makes you feel good about yourself. You have something to tell you that you’ve done it good.

P14

Young people also liked how similar the streak rewards were to those in other apps that they used:

The language (“streaks”) makes it easy for kids to understand because it’s like other apps like Snapchat.

P8

Young people found the daily feedback on their drinking goals easy to understand and liked the large graphics. The feedback indicates the achieved percentage of daily drinking goals and provides an appropriate, encouraging message based on the percentage. A few participants reported that they would like to be able to see if they went over their daily drinking goal, for example, 110%.

There was less engagement with the weekly feedback among some young people who did not use this feature. A small number reported that the notification to complete the feedback was not obvious enough, and they did not see the prompt. Those that did use the weekly feedback found the personalized feedback messages and linked support pages interesting but reported that they were too text heavy and long:

There could be a few pictures in it, stick figures. [P15]More pictures would be useful, because how your body works is quite difficult anyway, showing the bladder has muscles that contract too much.

P2

##### Clinician Feedback

A total of 8 clinicians provided feedback on the app (see Table 2 for full details). Clinician feedback focused on the consistency of the app with the best practice guidelines for bladder training, how the app aligned with their own practice, data use and integration with medical records, and using the app as part of clinical care.

##### Clinical Use and Appropriateness

The app design and content aligned with the best practice guidelines for bladder training. Clinicians were positive about app functions and customizability:

I think that looks good, particularly being set up with a clinician. I think it’s really easy to use, I mean I’ve used it and I’m not tech savvy...They can set whatever reminder they want themselves. [Clinician 5]I think that’s really good. I like the fact [the reminders are] every two hours, you’ve got the days of the week on there, they can do their own things with it. That’s what I’d say, you need to drink regular, these are the times you need to drink, with your breakfast, on your way to school.

Clinician 4

The range and size of the drinking containers were appropriate and fitted with the estimates used in clinics. A small number of clinicians recommended adding a container for small bottles used by younger children; however, they all agreed that a custom container could be made if needed:

Oh that’s good! That’s quite good because it’s visual isn’t it, especially the water bottle and the can. The majority of children I see, the lunch time drop in, they’ve always got those plastic bottles.

Clinician 4

Clinicians liked the functionality of recording drinks by pulling the fluid level down. They felt that this interactive nature would appeal to young people:

That’s clever [pull down to drink] because you say I’ve got this glass and I only drank half of it then it is a way of reflecting that, yeah that’s nice...yeah that’s really neat, I like the dragging down.

Clinician 7

Overall, clinicians felt the app fitted very well with their clinical practice and the information they needed during appointments. Some reported that being able to record the type of drinks, such as fizzy drinks, caffeine, or milk, as well as their amount would be useful, as this could affect UI symptoms:

My only comment is I would like to know what the child has had to drink...[for example] caffeine or fizz...Because I say to the kids avoid fizzy, avoid caffeine, milk doesn’t count as a drink.

Clinician 5

During the discussion about the app functions, it was decided that the option to record this in a free text diary would meet the clinicians’ needs, as plotting this information on the charts would be highly complicated.

##### Data Use and Integration With Medical Records

All clinicians said that the URApp data would be of clinical use and relevance. Clinicians reported that it was challenging for patients to provide an accurate log of their drinking and toileting since their previous clinic visit and felt that an app would have more appeal to young people:

It’s difficult to get young people to engage with recording diary information, including frequency/volume charts, with something a bit more modern you’d get more engagement and more data. [Clinician 7]Some of them are very easily distracted and don’t do their diaries and things. Especially the bigger ones they got no excuses because they’ve got phones on them all the time haven’t they. So they’ve got no excuse for not recording it.

Clinician 3

Views on how best to integrate the data with medical records varied. Some clinicians said that being able to download the data would be the best option for them, whereas others said they would use the data for discussions in their clinic and take written notes. The most acceptable solution for all clinicians was a screenshot of the data that could be shared with the user’s permission during a clinic session:

We have to document everything anyway, I’d use it all. Would be good if you could have it on a printout because you could put it in your records... I’d like a picture of it, it can be scanned into the records.

Clinician 4

##### Patient Engagement

The feedback on anticipated patient engagement was optimistic. Clinicians felt that the interactive nature of URApp would appeal to young people, particularly the customizability and reward systems:

I think we would use it on every single child that came to our clinic, anyone that came to bladder training, we would direct them all to it and say this is part of it, download this app we’re not going to give you an appointment until you’ve got some data on it.

Clinician 7

Clinicians felt it would be especially beneficial to use with patients who were not making progress with their treatment or were not engaging with their treatment plans:

I’m already thinking of kids I could use this with. I’m just going through a load of telephone reviews, and nothing has changed for these kids. It’s just exhausting really. [Clinician 6]It’s sustainable and it’s something that will get their concentration. They like these sorts of things. Sitting in front of someone being nagged at all the time, if they can actually do the app themselves and tap in all the stuff, and hopefully there’s obviously research to show it does motivate people and keep them going.

Clinician 6

##### Final App Modifications

Findings from the user testing phase were inputted into the table of changes and synthesized to identify the key areas for modification. All potential changes were discussed within the research team and coded using the MSCW framework with an additional *no change* code. Decisions were made based on repetition of feedback, importance for app functioning, importance for behavior change, consistency with clinical treatment guidelines, and cost of the change. A total of 9 key modifications were identified. Table 4 provides a summary of the identified changes and the app function areas.

**Table 4:** Summary of the final app modifications.

App function area	App modification
Recording new drinks	Pop-up message to explain how to pull down the fluid level on first use
Adding new drinks	Making the option to change the time of drink more obvious
Daily diary	Free text option for recording notes
Settings/about	Add a PDF link to instruction manual
Background and information pages	Reformat and reduce amount of text
Wees	Change leak text to include incontinence pad users
Poos	Pop-up with more information on stool consistency
Task center	Pulsing red button on home page to make this more obvious
Drinking goal feedback	Show goal completion over 100% if the user has exceeded their daily drinking goal

## Discussion

### Principal Findings

URApp is the first smartphone app specifically designed to support young people with UI. User testing among young people with UI demonstrated that URApp is acceptable, usable, engaging, and potentially effective in supporting concordance with bladder training. Young people liked the design and style of URApp and felt that it was age appropriate. Younger participants expressed a desire to be able to personalize the design of URApp to a greater extent, for example, by changing the theme colors or having seasonal backgrounds. Young people found the app quick and easy to use and liked the interactive nature of recording drinks.

All participants found URApp to be helpful in managing their drinking and toilet schedules, with many requesting to continue using the app beyond the study. Several participants reported that they had been able to increase their drinking or maintain more regular drinking as a result of using URApp. These findings are encouraging and provide preliminary evidence that URApp could be a potentially effective solution for providing personalized support to young people to self-manage their bladder symptoms.

URApp was designed to be discreet, and the mandatory passcode ensures privacy, a feature that was highly valued by the young people who tested the app. Previous research had found that timer watches prompt unwanted attention from peers, creating a barrier to their use [8,15]. URApp provides discreet prompts through the phone user’s SMS text message notification sound and allows users to customize their reminder text.

Young people were positive about the reward functions in URApp and felt this would motivate them to keep using the app. This is important, as continued concordance with the timed drinking and toileting schedule is crucial for successful bladder training [13].

Clinicians thought that URApp could provide an age-appropriate solution to aid concordance with bladder training in young people and, therefore, could be used as an adjunct to treatment. The option to customize the reminder time and text was particularly commended, as this could be tailored to each individual patient. Clinicians also reported that being able to record the type of drink was beneficial, as certain types of drinks (eg, fizzy and caffeinated drinks) might have an adverse impact on bladder function in some patients.

### Strengths and Limitations

URApp was developed using a rigorous approach to intervention and app design, which is underpinned by the behavior change theory. Development methods were guided by Medical Research Council recommendations for the development and evaluation of digital interventions [27] and informed by the PBA to intervention development [29]. This is the optimal approach for developing digital health interventions to ensure their usability and acceptability [41].

The end user population was included throughout the development process. This means that URApp was centrally designed around user needs and feedback. Engaging with the app development team from the project’s outset ensured that the proposed design and functions of the app were feasible in terms of cost and delivery within the project timeline.

Although our results are encouraging, this work does have limitations. User testing was restricted to young people with English as a first language and with predominantly high levels of educational ability. Further testing and refinement of URApp is needed with young people from a range of educational, socioeconomic, and ethnic backgrounds.

In addition, young people included in the study had already engaged in treatment for UI, either through primary or secondary care. It is not clear if URApp would offer the same level of acceptability and utility to young people who had not yet engaged in treatment. This is an important area for further research, as the stigma of continence problems prevents many young people from seeking treatment.

### Conclusions

This study provides initial support for the acceptability and usability of URApp. The next stage is to test whether URApp is effective in aiding concordance with bladder training, and therefore, improve bladder symptoms and enhance well-being. URApp should be tested across a range of settings, including pediatric continence clinics, primary care, and schools (via school nurses). The cost-effectiveness of using URApp to support bladder training in primary and secondary care settings and in the community also needs to be examined. An interactive website [23] has been developed where users can download URApp at no cost (available for iOS and Android devices) and access resources to support young people with UI.

## Appendix

Multimedia Appendix 1 Core app functions to support bladder training and target behavioral change.

Multimedia Appendix 2 Behavior change techniques used in URApp.

Multimedia Appendix 3 Example of final prototype.

## Abbreviations

BCT: behavior change technique
MSCW: must have, should have, could have, would have
NIHR: National Institute for Health Research
PBA: person-based approach
PPI: patient and public involvement UI: urinary incontinence
