# Supplementary material for: A Smartphone App for Supporting the Self-management of Daytime Urinary Incontinence in Adolescents: Development and Formative Evaluation Study of URApp
Source: JMIR Pediatr Parent. 2021 Nov 15;4(4):e26212. doi: 10.2196/26212 (PMC8663506; doi:10.2196/26212)
Supplement: Multimedia Appendix 1 [file pediatrics_v4i4e26212_app1.docx]

Appendix 1: Core app functions to support bladder training and target behavioural change

| **URApp core function** | **Target behavioural change / clinical need** |
| --- | --- |
| Setting a daily drinking goal | - Work towards daily drinking goal - Increase drinking to meet recommended fluid intake |
| Recording fluid intake | - Increase fluid intake - Regular drinking |
| Reminders to drink | - Regular drinking throughout the day (avoid drinking large amounts at one time, e.g. before bed) |
| Reminders to go to the toilet | - Regular toileting to exercise and stretch the bladder muscles, and increase bladder capacity |
| Tracking daily drinking goal | - Monitor fluid intake and progress towards goal |
