# Supplementary material for: A Smartphone App for Supporting the Self-management of Daytime Urinary Incontinence in Adolescents: Development and Formative Evaluation Study of URApp
Source: JMIR Pediatr Parent. 2021 Nov 15;4(4):e26212. doi: 10.2196/26212 (PMC8663506; doi:10.2196/26212)
Supplement: Multimedia Appendix 2 [file pediatrics_v4i4e26212_app2.docx]

Appendix 2: Behaviour change techniques used in URApp.

| **BCTs** | **Implementation in URApp** |
| --- | --- |
| - Information about health consequences of the behaviour - Instruction on how to perform the behaviour | URApp prototype currently includes links to help and support pages on:   - How the bladder works and benefits sticking to a regular drinking and toileting schedule - Information on optimal voiding behaviour, e.g. toilet posture. - How to set a drinking goal - Managing your symptoms - Drinking barriers and solutions |
| - Action planning - Prompts/cues | Information is provided about planning performance of the frequency and regularity of drinking and toileting behaviours. The most important aspect of urotherapy is drinking at regular intervals. URApp allows users to set regular reminders to prompt them to drink and to use the toilet. These are currently set to a default value of every 2 hours but can be customised to suit each user. This corresponds with clinical advice about desired frequency of toilet visits (every 2-3 hours). |
| - Goal setting - Graded tasks - Commitment / behavioural contract - Review behaviour goals | During urotherapy, patients discuss goals for fluid intake and a ‘behavioural contract’ is agreed with their clinician. Goals are reviewed at each clinic based on whether there is an improvement in symptoms.  URApp allows the user to set a drinking goal of 6-8 glasses, with the minimum fluid volume of each drink being 50ml and the maximum being 250ml (the recommended amount of fluid intake for our target age group).  Users incremental changes towards the target drinking goal as they use the app e.g. they start off with smaller volumes of fluid in each of their 8 glasses and gradually increase the volume as their symptoms improve. |
| - Feedback on behaviour - General encouragement - Tailored personalised message - Discrepancy between current behaviour and goal | URApp currently provides feedback messages about drinking and toileting behaviour.  Daily feeback is provided on progress towards daily drinking goal, for example: ….  Users are asked to complete a weekly evaluation about their drinking and symptoms. They then receive tailored feedback based on their answers. This includes motivational messages, advice on helpful changes, and links to further information. |
| - Rewards | URApp provides daily and monthly rewards.  Users receive streak rewards for daily use of the app.  By completing tasks within the app (for example drinking one glass, reaching their goal, recording a toilet visit), the user collects stars. At the end of each month they are given a trophy based on the amount of stars collected. |
| - Self-monitoring of behaviour - Monitoring of behavior by others with feedback | URapp allows users to record toileting and drinking behaviour and self-monitoring of these behaviours using progress charts. Charts display users’ progress towards reaching their drinking goal and how this relates to toileting. A range of data can be added to the chart including; number of wees, wee size, leaks, poo accidents, poo consistency.  The availability of accurate data on drinking and toileting is crucial for clinicians to be able to review, and provide feedback on, their patients’ progress and inform clinical decision-making. |
| - Reduce negative emotions | URApp provides positive encouragement and support through tailored feedback. Links to support pages are included.  Users have the ability to record their mood in a diary function.  Affective symptoms are associated with more severe bladder symptoms and with poor concordance to treatment. It is important to keep track of these symptoms to see if there are relationships with bladder symptoms. |
| - Barrier identification | Links to common barriers for drinking and toileting are including in the support pages. For example how to manage bladder therapy at school. |
