# Supplementary material for: A Smartphone App for Supporting the Self-management of Daytime Urinary Incontinence in Adolescents: Development and Formative Evaluation Study of URApp
Source: JMIR Pediatr Parent. 2021 Nov 15;4(4):e26212. doi: 10.2196/26212 (PMC8663506; doi:10.2196/26212)

Appendix 3: Example of final app prototype


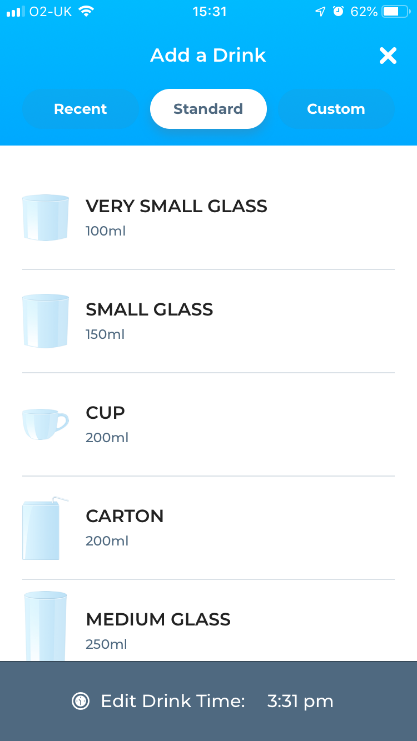

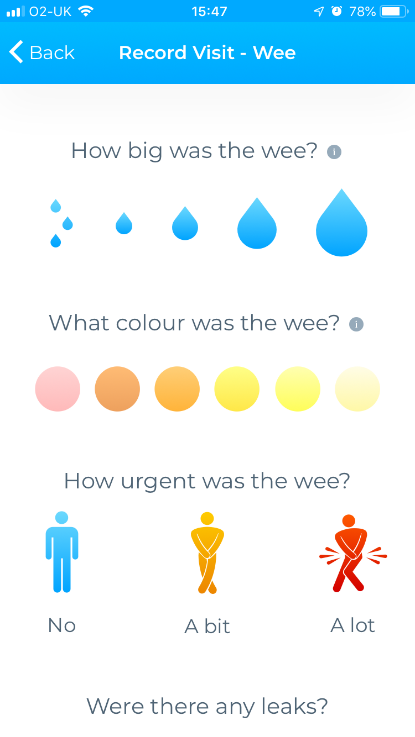

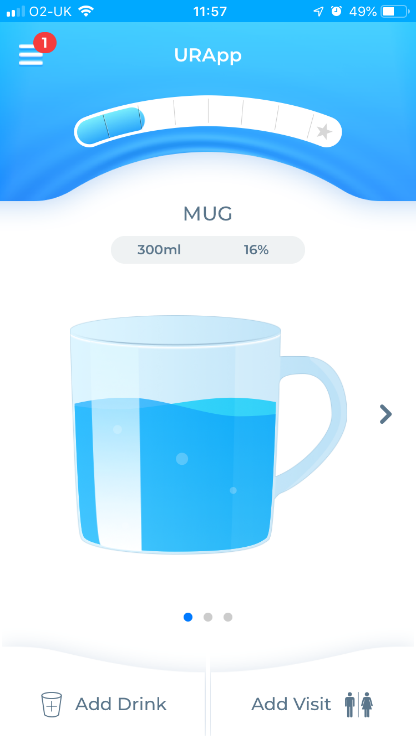
*Example of URApp prototype*

URApp rewards


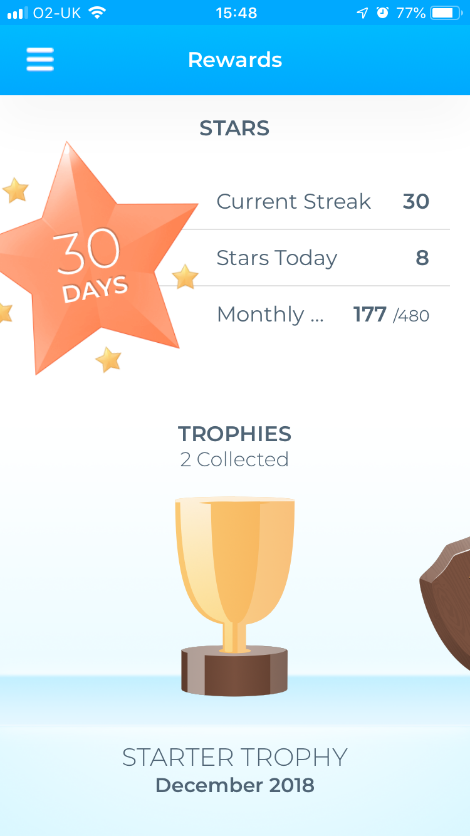

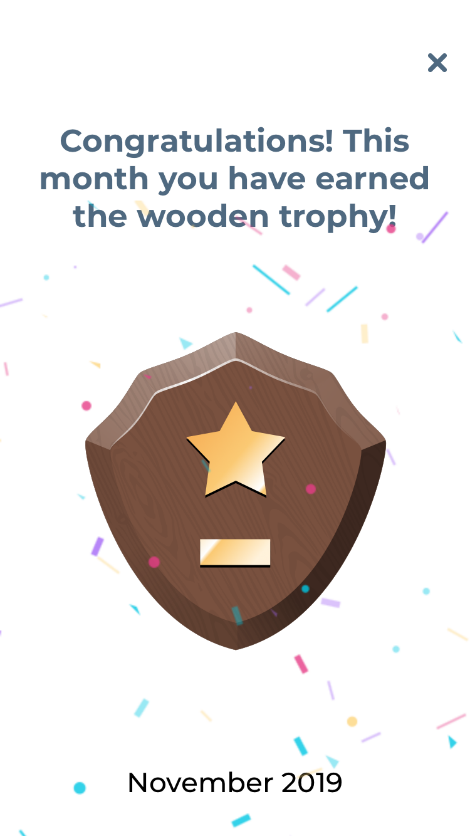


*Example of tailored feedback*


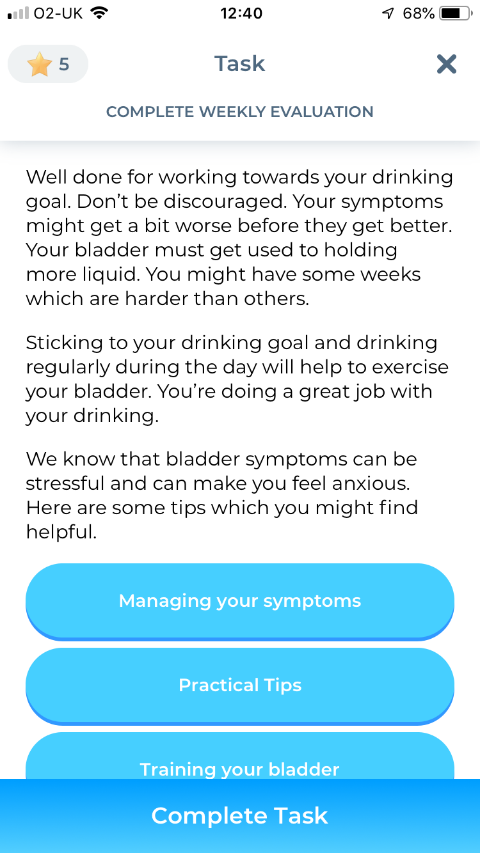

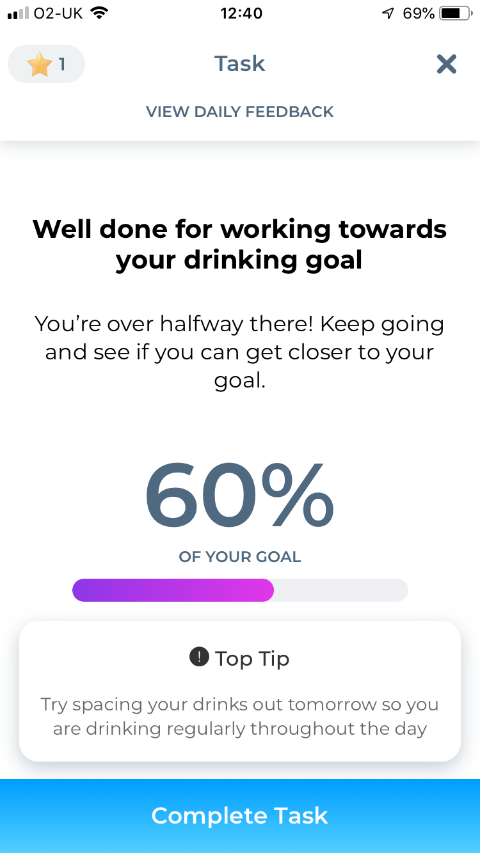

Supplement: Multimedia Appendix 3 [file pediatrics_v4i4e26212_app3.docx]
